# Colloidal HgTe Quantum Dot/Graphene Phototransistor with a Spectral Sensitivity Beyond 3 µm

**DOI:** 10.1002/advs.202003360

**Published:** 2021-02-01

**Authors:** Matthias J. Grotevent, Claudio U. Hail, Sergii Yakunin, Dominik Bachmann, Michel Calame, Dimos Poulikakos, Maksym V. Kovalenko, Ivan Shorubalko

**Affiliations:** ^1^ Department of Chemistry and Applied Biosciences ETH Zurich Vladimir Prelog Weg 1 Zurich CH‐8093 Switzerland; ^2^ Laboratory for Transport at Nanoscale Interfaces Swiss Federal Laboratories for Materials Science and Technology Überlandstrasse 129 Dübendorf CH‐8600 Switzerland; ^3^ Department of Mechanical and Process Engineering ETH Zurich Sonneggstrasse 3 Zurich CH‐8092 Switzerland; ^4^ Laboratory for Thin Films and Photovoltaics Swiss Federal Laboratories for Materials Science and Technology Überlandstrasse 129 Dübendorf CH‐8600 Switzerland; ^5^ Department of Physics University of Basel Klingelbergstrasse 82 Basel CH‐4056 Switzerland

**Keywords:** low‐temperature detectors, mercury telluride, photodetectors, phototransistors, QDs

## Abstract

Infrared light detection enables diverse technologies ranging from night vision to gas analysis. Emerging technologies such as low‐cost cameras for self‐driving cars require highly sensitive, low‐cost photodetector cameras with spectral sensitivities up to wavelengths of 10 µm. For this purpose, colloidal quantum dot (QD) graphene phototransistors offer a viable alternative to traditional technologies owing to inexpensive synthesis and processing of QDs. However, the spectral range of QD/graphene phototransistors is thus far limited to 1.6 µm. Here, HgTe QD/graphene phototransistors with spectral sensitivity up to 3 µm are presented, with specific detectivities of 6 × 10^8^ Jones at a wavelength of 2.5 µm and a temperature of 80 K. Even at kHz light modulation frequencies, specific detectivities exceed 10^8^ Jones making them suitable for fast video imaging. The simple device architecture and QD film patterning in combination with a broad spectral sensitivity manifest an important step toward low‐cost, multi‐color infrared cameras.

## Graphical abstract

Colloidal quantum dot/graphene phototransistors offer a viable alternative to traditional infrared detector technologies owing to inexpensive synthesis and processing of QDs. Here, the spectral sensitivity is extended up to 3 µm with HgTe QD/graphene phototransistors presenting specific detectivities of 6 × 10^8^ Jones, and kHz operation demonstrating potential for fast video imaging.

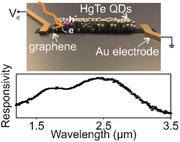

Night vision and passive imaging of thermal sources require infrared (IR) light detection with a spectral sensitivity up to 10 µm, while IR spectroscopy of gases and molecules require a spectral sensitivity ideally covering 2–15 µm. For these applications, highly sensitive, but costly, detectors are presently used; in particular, epitaxially grown narrow bandgap semiconductors such as HgCdTe and InGaAs alloys. For camera applications, the detector material is commonly grown on a separate substrate and later integrated into silicon‐based read‐out electronics by bump bonding, which is challenging for pixel pitches below 10 µm as it requires high assembling accuracy.^[^ ^1^, ^2^ ^]^ Furthermore, a thermal expansion coefficient mismatch between some materials and the read‐out electronics induces strain in the bump bonds resulting in a low reliability upon repetitive cryogenic cooling, the cooling is generally being required for photodetectors with spectral sensitivities beyond a wavelength of 3 µm.^[^ ^1^, ^2^, ^3^ ^]^ Therefore, bump bonding is a complex technical process, which increases the overall camera fabrication costs impeding the use in emerging applications such as in self‐driving vehicles. Other emerging technologies such as micro‐spectrometer and multi‐color (hyperspectral) IR cameras can be used for environmental monitoring, detection of hazardous gases, and for increased object identification making use of material specific spectral absorption, however, patterning of the IR active material is required.^[^ ^4^, ^5^ ^]^ In multi‐color cameras, each pixel can distinguish between multiple IR colors and first demonstrations of this include two‐color photodetectors based on stacked HgTe epitaxial thin films,^[^ ^4^ ^]^ HgTe QD thin films,^[^ ^6^ ^]^ or hybrid HgTe QDs on silicon.^[^ ^7^ ^]^ However, the integration of additional colors into these devices drastically increases the fabrication complexity. Alternatively, multi‐color IR cameras can be fabricated by dividing each pixel into color‐sensitive sub‐pixels^[^ ^8^ ^]^ requiring microfabrication and patterning techniques.

A device concept, based on colloidal QDs sensitized graphene field‐effect transistors (FETs),^[^ ^9^ ^]^ simultaneously provides high spectral sensitivities and potentially reduced overall detector costs by combining low‐cost fabrication with patterning techniques such as stamp transfer,^[^ ^10^ ^]^ and nanoprinting.^[^ ^11^, ^12^ ^]^ These phototransistors do not require bump bonding and can readily be integrated into silicon‐based read‐out electronics.^[^ ^13^ ^]^ High sensitivities have been reported for room‐temperature operation of PbS QD/graphene FETs with a spectral sensitivity determined by the QD size resulting in a spectral cutoff between 1000 and 1600 nm.^[^ ^9^ ^]^ While expanding the spectral range toward shorter wavelength has been demonstrated by employing QD materials such as ZnO^[^ ^14^, ^15^ ^]^ for UV, and perovskite QDs^[^ ^16^, ^17^ ^]^ or CdSe QDs^[^ ^18^ ^]^ for visible light detection, it has remained challenging to extend the spectral sensitivity to longer wavelengths, namely the short‐ and mid‐wavelength‐IR spectral range. For this task, HgTe QDs, with their tunable spectral sensitivity from short‐wavelength into terahertz range, are among the most suitable candidate materials.^[^ ^19^, ^20^, ^21^ ^]^ Various HgTe QD based photodetectors have been demonstrated with spectral sensitivities in the short‐wavelength and mid‐wavelength infrared region.^[^ ^22^, ^23^, ^24^, ^25^, ^26^, ^27^, ^28^, ^29^, ^30^, ^31^ ^]^ Some photoconductors and photodiodes have implemented the use of graphene electrodes (which is significantly different as compared to graphene field‐effect transistor devices) demonstrating responsivities in the low mA W^−1^ range.^[^ ^32^, ^33^ ^]^ Higher responsivities of 10^5^ A W^−1^ were reached with phototransistors based on HgTe QDs and MoS_2_ FETs up to a wavelength of 2.1 µm, but the phototransistor required an intermediate TiO_2_ buffer layer in between the HgTe QDs and the MoS_2_ to protect the MoS_2_ transistor and to generate a *pn*‐junction at the HgTe QD/TiO_2_ interface.^[^ ^34^ ^]^ In fact, the interface between graphene (or MoS_2_) and HgTe QDs is critical for achieving functional phototransistors, and the development and fabrication of HgTe QD/graphene phototransistors operating beyond 2.1 µm remain unprecedented.

Here, we demonstrate the fabrication and evaluation of HgTe QD/graphene phototransistors with high responsivities, and a specific detectivity of 6 × 10^8^ Jones at a wavelength of 2.5 µm, extending the spectral sensitivity beyond 3 µm. Furthermore, the phototransistor reveals operation at kHz light modulation frequencies with potential for fast video imaging.

**Figure** **1**a shows the fabrication process starting with a graphene FET made out of commercially available, chemical vapor deposited graphene (see Experimental Section). After the graphene FET was produced, HgTe QDs were selectively deposited on top of the graphene channel by electrohydrodynamic nanoprinting (see Experimental Section).^[^ ^11^, ^12^ ^]^ The HgTe QDs were synthesized following the protocol of Shen et al. to obtain non‐aggregated QDs, which is paramount for avoiding clogging of the printing nozzle.^[^ ^20^ ^]^ Transmission electron microscopy of a typical synthesis showed a spherical QD shape with QD diameters of about 5 nm (Figure S1a, Supporting Information). A powder X‐ray diffraction pattern exhibits a zinc‐blende crystal structure (Figure S1b, Supporting Information) in agreement with literature.^[^ ^20^, ^35^ ^]^ Nanoprinting, as a deposition method, enables a precise deposition of QDs on top of the graphene channel (5 µm width, 50 µm length). To prevent potential parallel conductance from one gold electrode through the narrow bandgap semiconductor film to the second gold electrode, the QD film was deposited on a smaller area to avoid overlap with the gold electrodes. As a result, the photoactive area is of a smaller dimension (5 × 40 µm) compared with the graphene transistor. For calculations, the full area of graphene (5 × 50 µm) was taken into account, as noise originates over the complete graphene area. After the nanoprinting, the pristine QDs ligands were exchanged with shorter 1,2‐ethanedithiol ligands by a single‐step solid‐state ligand exchange (see Experimental Section) resulting in a QDs layer thickness reduction from 410 to 340 nm as determined by atomic force microscopy (Figure S2, Supporting Information). The ligand exchange may not replace all pristine ligands with 1,2‐ethanedithiol, but it is sufficient enough to result in a functional phototransistor device. Further optimization of this device may be achieved by optimizing the QD layer thickness as it has been previously reported for PbS QD/graphene phototransistors.^[^ ^11^ ^]^ The phototransistor was electro‐optically characterized by a responsivity setup with modulated light (see Experimental Section). Figure 1b shows a top‐view optical bright‐field image of the device, and Figure 1c presents the spectral dependence of the phototransistor's responsivity operating at a temperature of 80 K. A first excitonic absorption feature is observed at a wavenumber of 4000 cm^−1^, which is red‐shifted by about 75 meV as compared to a Fourier‐transform infrared spectrometer (FTIR) absorption measurement (Figure S3, Supporting Information). The responsivity measurement differentiates from the FTIR measurement in terms of employed ligands for the QD film and the measurement temperature, which both can result in a bathochromic shift. Further extension of the spectral sensitivity into the long wavelength IR can be achieved by employing larger HgTe QDs.^[^ ^20^ ^]^

**Figure 1:**
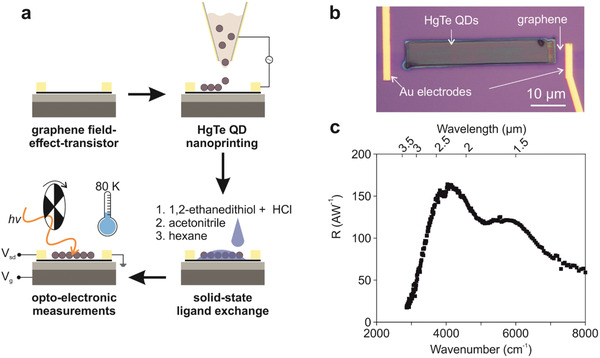
A HgTe QD/graphene phototransistor. a) Illustration of the fabrication process. b) Optical bright‐field image of the final device in a top‐view. c) Measured spectral dependence of the responsivity at a temperature of 80 K.

**Figure** **2**a presents the phototransistor responsivity as a function of the temperature and gate‐voltage (Figure S4, corresponding phase plot in the Supporting Information). At room temperature, the device does not exhibit any measurable photoresponse, but a photosignal appears at temperatures below 100 K and gate voltages above zero with a responsivity of about 800 A W^−1^ (at an applied drain bias of 0.5 V) at a temperature of 80 K which may even increase at lower temperatures and higher applied drain bias. In contrast to commercial IR detectors, the HgTe QD/graphene phototransistor can be fabricated directly on silicon‐based substrates. Furthermore, the HgTe QD/graphene phototransistor can be repetitively thermally cycled for at least 50 cycles between 300 and 80 K without device degradation nor failure (Figure 2b), and pixel pitches below 10 µm are within reach. The soft nature of the QD thin film likely reduces the likelihood of QD film ruptures, and even in the case of a QD film rupture, it would not lead to immediate device failure as photoinduced charge carriers are transported vertically to the graphene interface unaffected by vertical cracks. The photosignal is measured in the electron‐conducting regime of graphene (Figure 2c), under illumination, an increase of the current is measured illustrating the transfer of photoinduced electrons from the QD thin film to graphene (Figure S5, Supporting Information). Based on the similar device architecture of the HgTe QD/graphene phototransistor compared to other QD/graphene phototransistors, we assume a similar device mechanism.^[^ ^9^, ^10^, ^36^ ^]^ The Fermi levels of graphene and the HgTe QD layer align when brought into contact, resulting in a built‐in potential as schematically shown in Figure 2d. Under illumination with photon energies above the bandgap, electron–hole pairs are created and, subsequently, the charge carriers are separated at the interface with graphene. Holes are accumulated in the QD film while electrons are transferred to graphene changing the charge carrier conductivity in graphene. After the illumination is switched off, the charge carriers recombine possibly by a temperature‐driven Schottky‐like potential barrier hopping restoring the steady‐state configuration in dark. The likely dependence of the photosignal on the band alignment may result in the local responsivity maxima, for this device, around a gate voltage of about 9 V as observed in Figure 2c. Contributions from other mechanisms such as a bolometric effect would lead to a gate voltage independent photosignal, which could not be observed in our case (Figure 2c). Therefore, bolometric contributions to the overall photosignal can be neglected.^[^ ^37^, ^38^ ^]^

**Figure 2:**
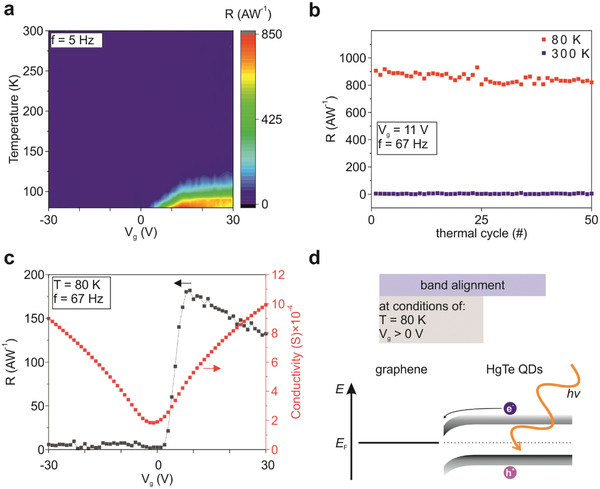
Electro‐optical characterization of the HgTe QD/graphene phototransistor. a) Measured temperature‐ and gate voltage‐dependent responsivity. b) Measured responsivity over a repeated number of thermal cycles. c) Measured gate‐dependent conductivity of graphene and responsivity at a temperature of 80 K. d) Schematic of the band alignment with photoinduced charge carrier transfer to graphene at a temperature of 80 K. The measurements were obtained at a drain bias of 0.5 V, a wavelength of 2.5 µm, and an irradiance of 1.5 × 10^−5^ W cm^−2^.

The photoresponse at low temperatures was observed to be quenched for perovskite QD/graphene phototransistors in a temperature range of 300–200 K.^[^ ^39^ ^]^ Surprisingly, this is not the case for our HgTe QD/graphene or for PbS QD/graphene phototransistors.^[^ ^40^ ^]^ Recently, we characterized PbS QD/graphene phototransistors at low temperatures and proposed a temperature‐dependent parallel shift of the QD conduction‐ and valence bands impacting the temperature‐dependent performance of the phototransistor.^[^ ^40^ ^]^ For example, the dependence of the energy levels on QD ligands is known to be reliant on variations of the QD surface dipoles and, so far, reported at a constant temperature.^[^ ^41^, ^42^, ^43^, ^44^, ^45^, ^46^ ^]^ The QD‐surface/ligand distance should be temperature‐dependent, and therefore, the QD surface dipoles as well, affecting the energy levels of the QD thin film.

**Figure** **3**a compares the photoresponse with noise measurements as a function of the gate voltage. The highest current‐normalized responsivity is achieved at a gate voltage of 8 V and declines at higher gate voltages, while the current‐normalized noise‐current increases at gate voltages above 0 V. Normalizing both values to the active phototransistor area gives the specific detectivity according to

(1) $$D^{*}=\frac{\frac{R}{I_{\mathrm{ds}}}\times \sqrt{A}}{\frac{i_{n}}{I_{\mathrm{ds}}}}=\frac{\sqrt{\mathrm{BW}\times A}}{\mathrm{NEP}}$$

where BW is the measurement bandwidth, NEP is the noise equivalent power, *A* is the device area (cm^2^), *R* is the responsivity (A W^−1^), *i*_n_ is the noise current (A Hz^‐1/2^), and *I*_ds_ the drain current. The specific detectivity D* is shown in Figure 3b with the highest value of 6 × 10^8^ Jones at a gate voltage of 6 V, light modulation frequency of 67 Hz, and a wavelength of 2.5 µm.

**Figure 3:**
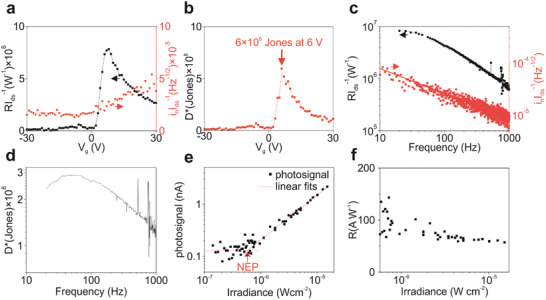
Comparison of responsivity and noise measurements. a) Responsivity and noise, and b) specific detectivity are plotted as a function of the gate voltage. c) Responsivity and noise, and d) specific detectivity are plotted as a function of the light modulation frequency. e) The irradiance dependence of the photosignal is shown, and f) the irradiance dependence of the photosignal in units of responsivity. The measurements were obtained at a temperature of 80 K, drain bias of 0.5 V, a wavelength of 2.5 µm, an irradiance of 1.5 × 10^−5^ W cm^−2^, gate voltage of 11 V, and a light modulation frequency of 67 Hz, unless the parameter was investigated in the respective measurement.

Both the responsivity and noise are strongly dependent on the light modulation frequency as shown in Figure 3c, with rise and fall times of the photosignal of about 0.4 and 0.7 ms, respectively (Figure S6, Supporting Information). The measured current‐normalized noise‐current declines at higher frequencies following a 1/f behavior neglecting the contributions from thermal noise and shot noise (see Noise Contributions in the Experimental Section). The responsivity decreases slowly up to 50 Hz and declines faster beyond 50 Hz, and a 3 dB bandwidth (50% of the highest responsivity) is reached at 110 Hz. The small differences in the decline of the frequency behavior of the responsivity and noise are more present in Figure 3d. The frequency dependence of the specific detectivity reaches 2.9 × 10^8^ Jones at a gate voltage of 11 V and a frequency of 45 Hz, and decreases at higher frequencies. While the frequency bandwidth is defined as a 50% drop in the responsivity value, more attention should be given to the bandwidth of the specific detectivity. Phototransistors exhibit high 1/f noise behavior which compensates the responsivity drop to some extent, while photodiodes have a flat noise behavior and a decrease in responsivity leads to the same decrease in the specific detectivity. Here, 50% of the maximum specific detectivity is still achieved at 700 Hz and proves that these hybrid HgTe QD/graphene phototransistors can be operated at moderate frequencies at the cost of lower responsivity. When larger responsivities are required at low light intensities for small area devices, the drain bias can be increased which will simultaneously increase the responsivity and noise current, but leaves the specific detectivity unaffected within an ohmic operational range. Figure 3e illustrates the photosignal of the phototransistor as a function of the irradiance, while Figure 3f shows the corresponding responsivity dependence, both obtained at a gate voltage of 11 V. By reducing the light intensity down to a signal‐to‐noise ratio of unity, the noise equivalent power (NEP: 1.4 × 10^‐12^ W) is derived as marked in Figure 3e. This is an alternative method to measure the specific detectivity according to Equation (1), resulting in 3.5 × 10^8^ Jones with an equivalent noise bandwidth of 0.094 Hz (defined by the lock‐in settings), which is highly similar to the value calculated from the responsivity and noise measurements obtained under the same measurement conditions (2.9 × 10^8^ Jones).

The demonstrated HgTe QD/graphene phototransistor device concept is accompanied by three main advantages (i) the spectral sensitivity can be tuned by the QD material, (ii) the phototransistor can be patterned by precise, local deposition of the QDs, as required for multi‐color photodetectors, and (iii) the phototransistor can directly be fabricated on silicon‐based substrates with robust phototransistor performance upon thermal cycling. Such a HgTe QD/graphene phototransistor shows an inferior specific detectivity compared to commercial photon detectors,^[^ ^47^ ^]^ but there is an ample space to further optimize the HgTe QD/graphene phototransistors. For example, an improved specific detectivity is expected by QD deposition over the complete graphene channel, and at an optimized QD layer thickness. Furthermore, it is likely that the temperature‐dependent responsivity can be improved by employing different ligands, and optimized QD/ligand combinations may raise the operational temperature improving the specific detectivity. For example, a solution‐based ligand exchange with a combination of organic and inorganic ligands (2‐mercaptoethanol, HgCl_2_), followed by QD deposition leads to HgTe QD films with higher charge carrier mobilities.^[^ ^27^, ^28^ ^]^ The latter leads to longer charge carrier diffusion‐length which is beneficial for the extraction of photoinduced charge carriers out of thicker QD films and likely results in an improved device operational speed. Furthermore, the conduction‐ and valence band positions can be modified by varying the amounts of HgCl_2_ in the ligand exchange procedure,^[^ ^28^ ^]^ and can lead to an optimized band alignment with graphene.

In summary, by employing HgTe QDs, the spectral sensitivity of QD/graphene phototransistors has been extended to midwavelength‐IR spectral range up to 3 µm. The phototransistor shows no photosignal at room temperature, but at 80 K a specific detectivity of at least 6 × 10^8^ Jones at a wavelength of 2.5 µm and a frequency of 67 Hz is obtained. The specific detectivity declines by 50% at moderate light modulation frequencies of 700 Hz, well suitable for fast video recording. Higher specific detectivities may be achieved by increasing the QD layer coverage on graphene, optimization of the QD layer thickness, or utilizing different ligands and, by that, raising the operational temperature that optimizes the responsivity. Extending the spectral sensitivity of QD/graphene phototransistors into the midwavelength IR is a major advance towards fast multi‐color phototransistor arrays, which can benefit from the simple device architecture by fabrication of the phototransistors on a graphene FET platform, local QD deposition, and spectral sensitivity determined by the QD material.

## Experimental Section

##### Chemicals

Acetonitrile (≥ 99.9% Fisher Chemical); Ammonium persulfate (≥ 98.0% Sigma Aldrich); bis(trimethylsilyl)telluride (98%, Acros Organics); 1,2‐ethanedithiol (≥ 98%, Sigma Aldrich); hexane (≥ 97.0%, Sigma Aldrich); anhydrous hexane (97%, Acros); hydrochloric acid (37%, VWR Chemicals); mercury(II) chloride (≥ 99.5%, Sigma Aldrich); anhydrous methanol (99.8% PanReacAppliChem); oleylamine (> 95%, Strem Chemicals); anhydrous tetrachloroethylene (≥ 99%, Sigma Aldrich); Tetradecane (≥ 99%, Sigma Aldrich). All chemicals were used as received, except otherwise stated. Tetradecane and oleylamine were dried and degassed under reduced pressure at a temperature of 120 °C for 1 h before their use.

##### HgTe QDs Synthesis

(*Caution*: Highly toxic materials and heavy metals are used and appropriate precautions have to be implemented.) The HgTe QDs were synthesized following a protocol by Shen et al.^[^ ^20^ ^]^ Briefly, 27 mg (0.1 mmol) HgCl_2_ were dissolved in 4 mL dried oleylamine at 100 °C for 1 h under inert atmosphere. A mixture of 15 µL (0.05 mmol) of bis(trimethylsilyl)telluride in 0.5 mL anhydrous hexane was injected into the Hg^2+^ containing solution. After 1 min of QDs growth at 100 °C, the dispersion was quenched in 4 mL tetrachloroethylene. The QDs were precipitated from the dispersion by adding 3.6 mL methanol, centrifuged at 12 100 rpm for 1 min and the precipitate was dispersed in 0.9 mL tetrachloroethylene. The HgTe QDs were precipitated a second time with 0.5 mL methanol and centrifuged 12 100 rpm for 1 min. A small amount of the precipitate was dispersed in tetrachloroethylene, filtered through a 0.2 µm polytetrafluoroethylene syringe filter. The dispersion in tetrachloroethylene has been used to fabricate HgTe QDs thin films and measure their absorbance with Fourier‐transform infrared spectroscopy. The main precipitate was dispersed in 0.5 mL dried tetradecane and filtered through a 0.2 µm polytetrafluoroethylene syringe filter. The QDs dispersion was stored under dry and inert atmosphere.

##### Graphene Field‐Effect Transistor Fabrication

Commercially available monolayer graphene (“Easy Transfer,” Graphenea Inc.) grown by chemical vapor deposition was transferred on to a highly *p*‐doped silicon substrate with chlorinated 285 nm dry thermal SiO_2_ following a wet chemical transfer process. The graphene channel was defined by electron beam lithography, electron beam assisted thermal deposition of 70 nm thick Cu protective layer, and subsequent oxygen plasma etching for 150 s (200 W, 1 mbar O_2_). While the graphene was etched beside the Cu, it remained intact underneath the Cu. In a next step the Cu was etched with a water‐based ammoniumpersulfate solution (1.6 mg mL^−1^) for 5 min, and the electron beam lithography was repeated followed by an electron beam assisted thermal deposition of 2 nm Cr and 40 nm Au to form the electrodes.

##### Ligand Exchange

After nanoprinting of the HgTe QDs, the native oleylamine ligands were exchanged with 1,2‐ethanedithiol in a single‐step solid‐state ligand exchange at ambient conditions. A drop of a ligand exchange solution (10 mL acetonitrile, 200 µL HCl_(aq)_, 200 µL 1,2‐ethanedithiol) was placed on the sample for about 7 s, removed by spinning the sample and rinsed with acetonitrile, followed by hexane. After the ligand exchange had been performed, the sample was stored in an air‐ and water‐free atmosphere.

##### Responsivity Setup

Light from a globar (SLS203L, Thorlabs) was focused with a CaF_2_ lens, and modulated with a chopper (MC2000B‐EC, Thorlabs), onto a monochromator (SpectraPro HRS‐300, Princeton Instruments) equipped with two gratings (150 G mm^−1^, blaze wavelength of 0.8 µm; 150 G mm^−1^, blaze wavelength of 2 µm). Higher frequency orders were filtered out with longpass filters (780, 1000, 1500 nm, Ge wafer). The light was collimated with a CaF_2_ lens, mounted on a stage to adjust for the wavelengths‐dependent focal lengths. The collimated light was split into two beams with a 50/50 polkadot beamsplitter. One beam illuminated a reference detector (UM‐9B‐L, gentec‐eo) mounted behind a CaF_2_ window and connected to a lock‐in (SR860, Stanford Research Systems). The other beam illuminated the sample, in equal distance to the beam splitter as compared with the reference detector, mounted in a cryostat (ST‐100, JANIS) with CaF_2_ windows and an operating temperature range between 80–300 K. The sample was biased with a source‐measurement unit (2614B, Keithley), and the photosignal was measured as voltage fluctuations with a lock‐in (SR865, Stanford Research Systems) over a 100 Ω load resistor connected in series with the sample. A gate voltage was applied with a source‐measurement unit (2614B, Keithley) and the measured gate leakage did not exceed 10 nA. Measurements were performed at reduced pressure (10^−5^ hPa).

##### Noise Setup

A battery‐powered SR570 current amplifier was used to bias the sample and to amplify the current. The current was recorded with a NI USB‐6281 data acquisition board at a sampling rate of 625 kHz, and a 40 kHz lowpass filter. The signal was band‐limited with a tab 100 impulse response digital filter to 78 kHz and downsampled by a factor of four. Ten time‐traces each one second long were recorded and the resulting power spectral densities were averaged. The 50 Hz frequency was manually removed from the frequency spectra. Measurements were performed at reduced pressure (10^−5^ hPa).

##### Nanoprinting

We followed a previously published protocol.^[^ ^11^ ^]^ Briefly, the graphene FET was grounded over a 10 kΩ resistor. The HgTe QD dispersion with a concentration of about 10 mg mL^−1^ was filtered through a 0.1 µm polytetrafluoroethylene syringe filter and backfilled in a gold‐coated printing nozzle, with an orifice diameter of about 2 µm. The printing nozzle was placed 6–7 µm above the sample, and small droplets were ejected from the printing nozzle by applying a square wave voltage pulse (290 V, 1 kHz) between the nozzle and the sample. The nanoprinting was performed under ambient conditions at 25 °C and 35–40% relative humidity.

##### Noise Contributions

The lowest noise level was around 3.8 × 10^‐10^ A Hz^‐0.5^ in the 1/f noise spectrum at a modulation frequency of 1 kHz (taking a drain current of 3.8 × 10^−5^ A at a drain bias of 0.5 V). At the same measurement conditions, the calculated thermal noise was 1.1 × 10^−12^ and 0.6 × 10^−12^ A Hz^−0.5^ at 300 and 80 K, respectively; and the calculated Shot noise was about 3.5 × 10^−12^ A Hz^−0.5^. Therefore, the thermal and shot noise contributed together about 1% or less to the total noise level and their influence could be neglected.

## Conflict of Interest

The authors declare no conflict of interest.

## Author Contributions

M.J.G., I.S., M.V.K. conceived and designed the experiments. M.J.G. fabricated the graphene FETs and carried out the optoelectronic measurements and AFM measurements. M.J.G. synthesized the HgTe QDs. The QDs were nanoprinted by C.U.H. with contributions to the experimental design and discussions with D.P. The noise measurements were conducted by D.B. with assistance of M.C. The paper was written by M.J.G. with contributions by all authors and all authors have given approval to the final version.

## Supporting information

Supporting Information
