# Supplementary material for: Colloidal HgTe Quantum Dot/Graphene Phototransistor with a Spectral Sensitivity Beyond 3 µm
Source: Adv Sci (Weinh). 2021 Feb 1;8(6):2003360. doi: 10.1002/advs.202003360 (PMC7967065; doi:10.1002/advs.202003360)
Supplement: Supplementary file 1 — Supporting Information [file ADVS-8-2003360-s001.pdf]

© 2020 Wiley-VCH GmbH

ADVANCED SCIENCE
Open Access

Supporting Information

for *Adv. Sci.,* DOI: 10.1002/advs.202003360

Colloidal HgTe Quantum Dot/Graphene Phototransistor with a Spectral Sensitivity Beyond 3 µm

Matthias J. Grotevent, Claudio U. Hail, Sergii Yakunin, Dominik Bachmann, Michel Calame, Dimos Poulikakos, Maksym V. Kovalenko, Ivan Shorubalko**

Supporting Information:

Colloidal HgTe Quantum Dot/Graphene Phototransistor with a Spectral Sensitivity Beyond 3 µm

Authors

Matthias J. Grotevent,[1,2] Claudio U. Hail,[3] Sergii Yakunin,[1,4] Dominik Bachmann,[2] Michel Calame,[2,5] Dimos Poulikakos,[3] Maksym V. Kovalenko,[1,4,] Ivan Shorubalko[2,]**

Affiliations

[1] Department of Chemistry and Applied Biosciences, ETH Zurich, Vladimir Prelog Weg 1, CH-8093 Zurich, Switzerland

[2] Laboratory for Transport at Nanoscale Interfaces, Empa—Swiss Federal Laboratories for Materials Science and Technology, Überlandstrasse 129, CH-8600 Dübendorf, Switzerland

[3] Department of Mechanical and Process Engineering, ETH Zurich, Sonneggstrasse 3, CH-8092 Zurich, Switzerland

[4] Laboratory for Thin Films and Photovoltaics, Empa—Swiss Federal Laboratories for Materials Science and Technology, Überlandstrasse 129, CH-8600 Dübendorf, Switzerland

[5] Department of Physics, University of Basel, Klingelbergstrasse 82, CH-4056 Basel, Switzerland

* Correspondence should be addressed to M.V.K. (E-mail: mvkovalenko@ethz.ch) or I.S. (E-mail: ivan.shorubalko@empa.ch)

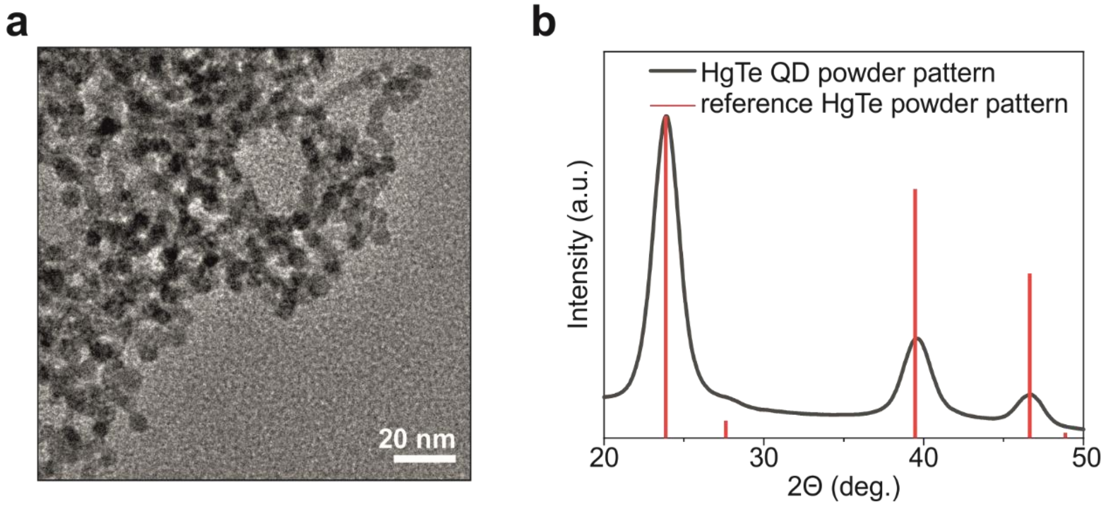

Figure S1. Analysis of a typical HgTe QD synthesis. (a) Transmission electron microscopy image of HgTe QDs. (b) Powder X-ray diffraction pattern (in black) of HgTe QDs, with a HgTe powder pattern reference (639255-ICSD)[1] in red. The transmission electron microscopy images were measured with a Tecnai F30 (Thermo Fisher Scientific) operated at an acceleration potential of 300 kV (field emission gun). The powder XRD measurement was obtained with a Stoe&Cie IPDS II Diffractometer with an image plate detector, Cu-Kalpha radiation (graphite monochromator, lambda = 1.54186 Å) and with in-house modifications (beamstop, sample holder) for the measurement of nano-materials.

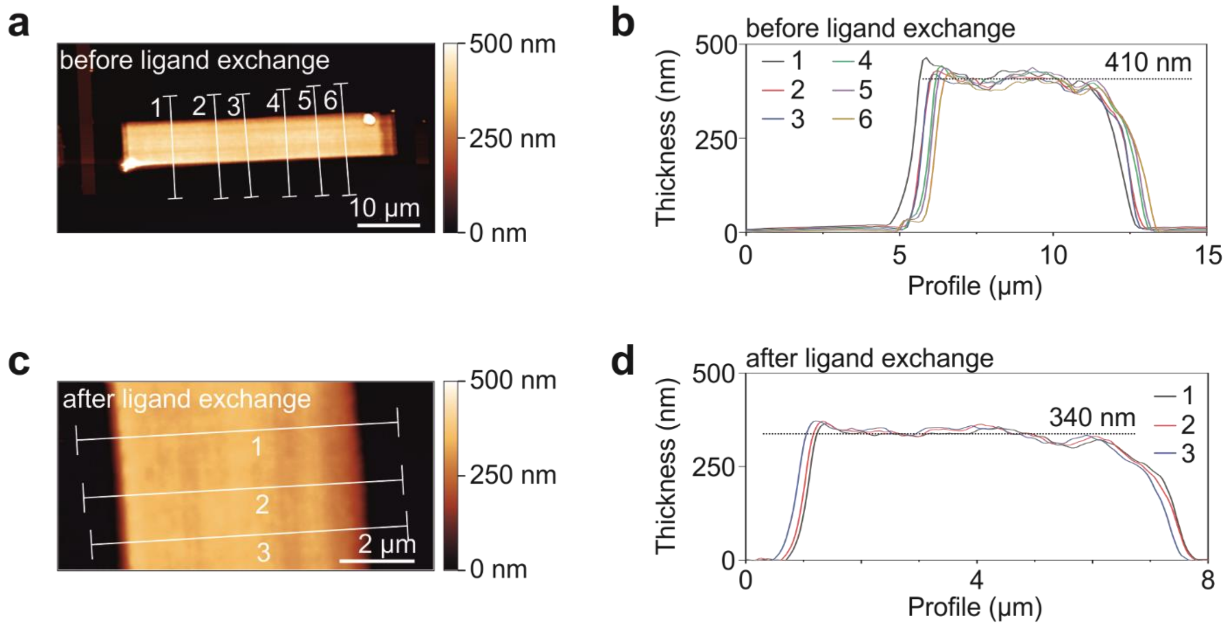

Figure S2. Atomic force microscopy image of the phototransistor (a) before ligand exchange with a resolution of 512×256 pixels, the corresponding cross-section profiles are plotted in (b); and in (c) after ligand exchange with a resolution of 5256×128 pixels, corresponding cross-section profiles are plotted in (d). The AFM images were recorded with a Bruker Dimension Fastscan in tapping mode.

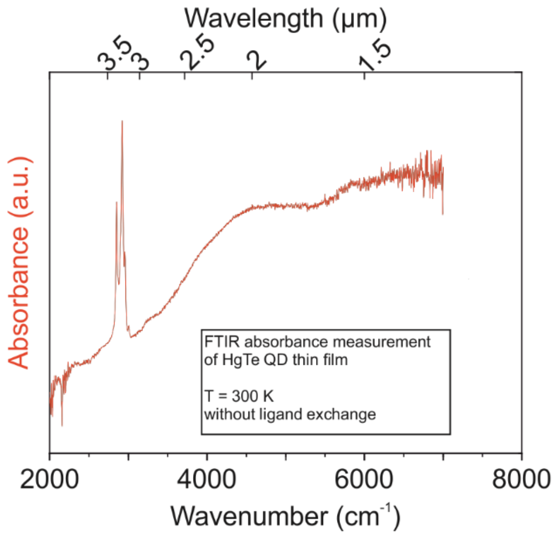

Figure S3. Absorbance of a HgTe QD thin film measured with a Fourier-transform infrared spectrometer (Nicolet 6700, Thermo Fisher Scientific). The double peak at 3.3 µm can be assigned to light absorption from the organic ligands of the QDs.

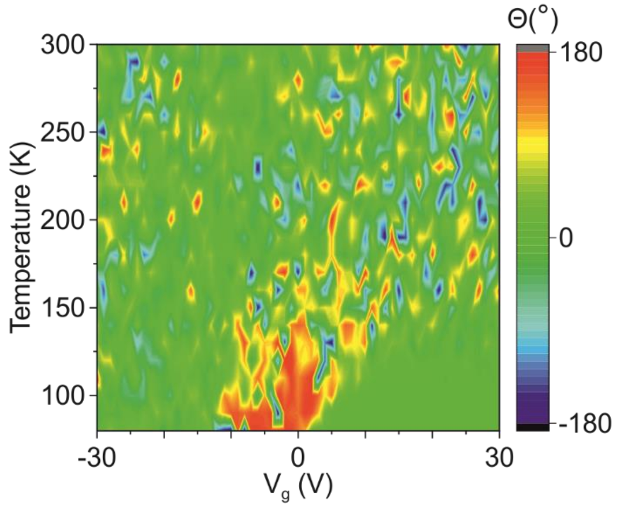

Figure S4. The phase of the responsivity measurement of the phototransistor.

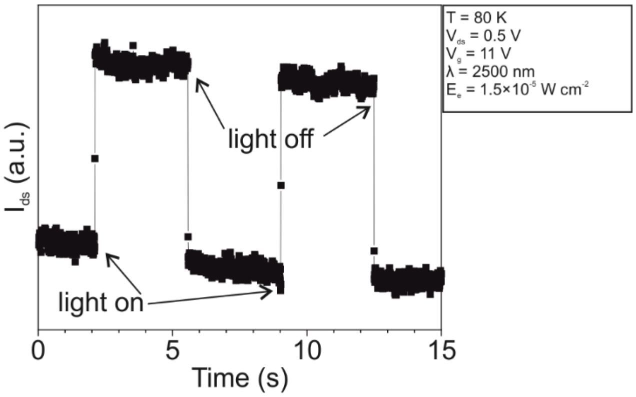

Figure S5. Time-trace of the HgTe QD/graphene phototransistor. Under illumination, the drain current increases, illustrating photoinduced electron transfer from QDs to graphene.

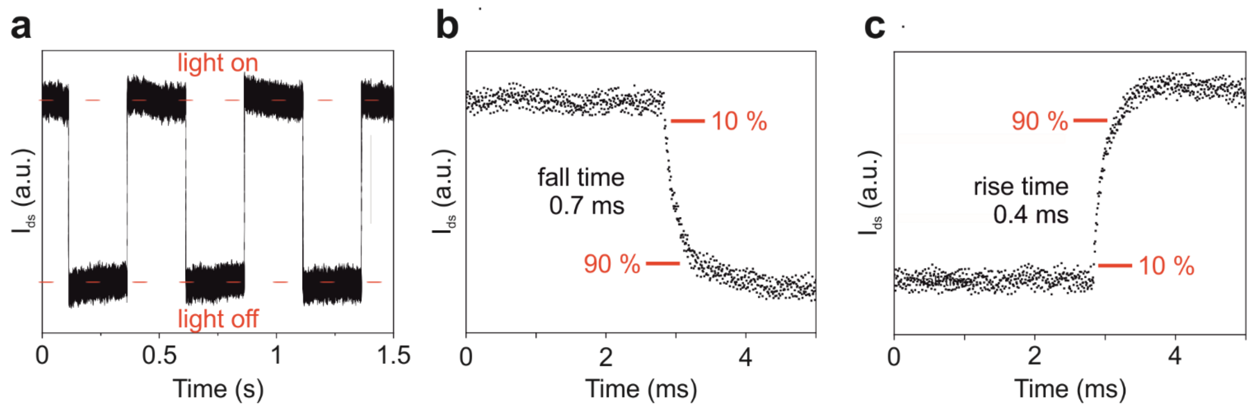

Figure S6. Time-trace of the HgTe QD/graphene phototransistor, with drain bias of 0.5 V, a gate voltage of 11 V, recorded with a NI USB-6281 data acquisition board while illuminated by a square-wave pulsed 1200 nm LED light source. (a) Shows an overview of the time-trace, (b) show the zoomed-in time-trace (with an x-axis offset of 0.610 s) for the fall time, and (c) shows the zoomed-in time-trace (with an x-axis offset of 0.860 s) for the rise time of the detector.

References

(1) Huang, T.; Ruoff, A. L. Pressure-Induced Phase Transitions of HgTe. *Phys. Status Solidi* **1983**, *193*, 93–95.
